# Management of Clinically Negative Neck in Early-Stage (T1-2N0) Oral Squamous-Cell Carcinoma (OSCC): Ten Years of a Single Institution’s Experience

**DOI:** 10.3390/jcm13237067

**Published:** 2024-11-22

**Authors:** Danilo Di Giorgio, Marco Della Monaca, Riccardo Nocini, Andrea Battisti, Federica Orsina Ferri, Paolo Priore, Valentina Terenzi, Valentino Valentini

**Affiliations:** 1 Department of Odontostomatological and Maxillofacial Sciences, La Sapienza University of Rome, 00185 Rome, Italy; danilo.digiorgio@uniroma1.it (D.D.G.);; 2 Unit of Otolaryngology, Head and Neck Department, University of Verona, 37134 Verona, Italy; 3 Department of Surgical Sciences, Tor Vergata University, 00133 Rome, Italy

**Keywords:** OSCC, early stage, oral cavity cancer, T1T2N0, neck management

## Abstract

**Background/Objectives**: Oral cavity squamous-cell carcinoma is among the most frequent head and neck neoplasms. Early-stage T1/T2N0 accounts for 40/45% of new diagnoses. Of these, about 30% of cases hide occult metastases in the neck. The management of clinically N0 neck is of paramount importance and is still being debated. **Methods**: The medical records of patients with a clinical diagnosis of early-stage T1-T2N0 carcinoma of the oral cavity between 2011 and 2021 were retrospectively analysed. The inclusion criteria were complete medical and radiological records, pT1-2 pathology staging, and a minimum follow-up of 24 months. Biographical, management, and survival data were analysed using IBM SPSS Statistics [28.0.1.1]; IBM Corp., Armonk, NY, USA). **Results**: A total of 121 patients met the inclusion criteria. The tongue was the most affected site, with 52 cases. All patients underwent resection of the primary tumour; for neck management, 47 (38.8%) underwent elective neck dissection, 36 underwent follow-up, and 11 underwent sentinel lymph node biopsy. A total of 59 cases were staged as T1 and 62 as T2; in 97 (80.2%) cases, the neck was confirmed as N0; in 10 (8.3%), N1; in 1 case, N2a; in 8, N2b; in 2, N2c; and in 3, N3b. The mean DOI was 4.8 mm. In a Cox regression, a statistically significant association was shown between overall survival and pN staging (*p* < 0.05). Kaplan–Meier analysis showed a statistically significant difference between different regimens of management of the neck in terms of overall survival, disease-free survival, and disease-specific survival in favour of elective neck dissection and sentinel lymph node biopsy compared to watchful policy (*p* < 0.05). **Conclusions**: Elective neck dissection and sentinel lymph node biopsy proved to be safe and oncologically effective in the treatment of clinically N0 early-stage oral carcinoma.

## 1. Introduction

Squamous-cell carcinoma of the oral cavity (OSCC) is a neoplastic disease with a potentially devastating impact on the quality of life of affected patients [1]. The estimated annual incidence is about 400,000 new cases worldwide—of which about 10,000 are registered annually in Italy—and a mortality rate of 178,000 cases [2]. All OSCCs are staged in accordance with the AJCC TNM (8th edition) classification system. Early stages are defined as T1-T2 cases, characterised by a primary tumour up to 4 cm in size and a maximum infiltration depth of up to 10 mm, as well as the absence of clear metastases to locoregional lymph nodes (cN0) [3]. About 45% of new diagnoses are clinically staged as an early stage (T1/T2N0), although about 30% of OSCCs conceal an occult neck metastasis [4,5]. If we consider that the presence of a single metastatic lymph node is associated with a 50% relative reduction in 5-year overall survival (OS), we understand how crucial proper management of the clinically negative neck (cN0) is in patient treatment [6]. Over the years, various techniques have been proposed for the management of clinically negative N0 neck, but to date, there has been no real gold standard defining the best treatment, and the choice remains controversial. The management of cN0 neck in patients with OSCC is considered one of the most controversial issues in head and neck oncology. The literature is divided between watchful policy consisting of follow-up (FU), elective neck dissection (END), and sentinel lymph node biopsy (SLNB). A large number of retrospective studies have been published in the literature both in favour of and against elective neck dissection [7,8]. Proponents of elective neck dissection base their position on data from retrospective studies suggesting that END results in better regional control and long-term survival. Opponents of elective neck dissection argue that it has no survival benefit and increases healthcare costs and donor site morbidity. Over the past two decades, SLNB has emerged as a potential alternative to END and FU in patients with early-stage OSCC, promising to replace END, under the right indications, while reducing costs to the healthcare system. The main attraction of SLNB for oral cancer is that it may allow patients to avoid the additional time and morbidity of END, allow for minimally invasive assessment of lymph node status in patients with OSCC, and detect potentially involved lymph nodes that are outside the expected lymphatic drainage patterns. However, the main problem with SLNB is the possibility of a false-negative diagnosis and, moreover, the need to refer the patient to a second surgical procedure of neck dissection in case of a positive result. Currently, the debate is still ongoing and there is no consensus on the best treatments for cN0 neck in patients with early-stage OSCC. As proof of this, major international multicentre randomised clinical trials are still ongoing, including the NGR-HN006 led by Prof. Lai of the MD Anderson Cancer Centre, now in phase II [9]. Based on the evidence presented thus far, it is evident that there is a need to continue data collection in order to ascertain which neck management treatments which have not yielded positive outcomes in clinical terms may nonetheless prove safer and more efficacious from an oncological standpoint, particularly in cases of early-stage OSCC, including those undergoing FU, END, and SLNB. The aim of this study was to analyse the experience of a single oncology institution in N0 neck management of early-stage OSCC cases over a 10-year period and to highlight statistically significant differences and associations in the various histological factors influencing prognosis and in the different treatment modalities for clinically negative neck.

## 2. Materials and Methods

All medical records of patients treated for oral cancer at the department of maxillofacial surgery, Umberto I general hospital, Sapienza University, Rome, Italy, from 2011 to 2021 were reviewed. The inclusion criteria were represented by a primary diagnosis of OSCC, pT1-2 staging, a complete clinical–radiological documentation, histology compatible with squamous-cell carcinoma, neck management including END, FU, or SLNB, and a minimum 24-month recorded follow-up. The exclusion criteria were pT3-4 and/or N+ clinical staging, histology other than squamous-cell carcinoma, perioral or nasal skin neoplasms with oral cavity invasion, and patients lost to follow-up before 24 months. For staging purposes, all patients underwent at least one pre-operative evaluation consisting of an objective examination, face and neck magnetic resonance imaging (MRI), cervical ultrasound, and chest computer tomography (CT). The main data collected were sex, age, site, T management, N management, type of reconstruction, grading, depth of invasion (DOI), perineural invasion (Pn), lymphovascular invasion (LV), any adjuvant therapies, and patient status. All patients were staged using the seventh TNM classification and staging of the American Joint Committee on Cancer for oral cancer. All statistical analyses were performed using IBM SPSS Statistics Software [28.0.1.1]; IBM Corp., Armonk, NY, USA. In addition to descriptive statistics, Cox proportional hazards regression and Kaplan–Meier curves were performed to assess the effect of the main histopathological parameters on survival in our patient cohort, while statistical analysis using the log-rank test (Mantel–Cox) was used to compare the different types of neck treatment with respect to patient survival, differentiating between overall survival, disease-specific survival, and disease-free survival. The null hypothesis of the statistical study was that the FU waiting policy was non-inferior to the END and SLNB surgical treatments in terms of efficacy and oncological safety. A *p*-value < 0.05 with a 95% confidence interval (CI) was chosen as the statistical significance level.

## 3. Results

Of the more than 600 patients screened, 121 met all study criteria and were enrolled. Among these, we recorded 65 men (53.7%) and 56 women (46.3%) with a mean age of 70.2 years, of whom 75% were over 60 years of age. Regarding sites, the tongue was the most frequent, with 52 cases (43%), followed by the inferior alveolar ridge and cheek mucosa, equiprevalent with 17 cases (14%), and the oral pelvis, with 14 cases (11.6%); less frequent were the other sites of the oral cavity, the upper alveolar ridge and retromolar trigone, both with 6 cases (5%); the lip and palate, with 4 cases each (3.3%); and the tongue extended to the soft palate, with 1 case (0.8%). From the clinical point of view, all patients were staged N0 following an objective examination and radiological examinations, while with regard to the primary tumour, 55 were clinically staged as T1 (46.5%) and 66 as T2 (54.5%). From the point of view of therapeutic management, all patients underwent T resection (100%); of these, 36 (29.8%) were subjected to a ‘wait and see’ policy, i.e., FU; 11 (9.1%) underwent SLNB, and 68 (56.2%) received END (Table 1). In this cohort of patients, 21 (17.2%) underwent contralateral neck dissection, of which 6 cases were deferred. Of the total number of patients who underwent resection and neck dissection, 40 received monobloc compartmental resection using the pull-through technique.

**Figure 1:**
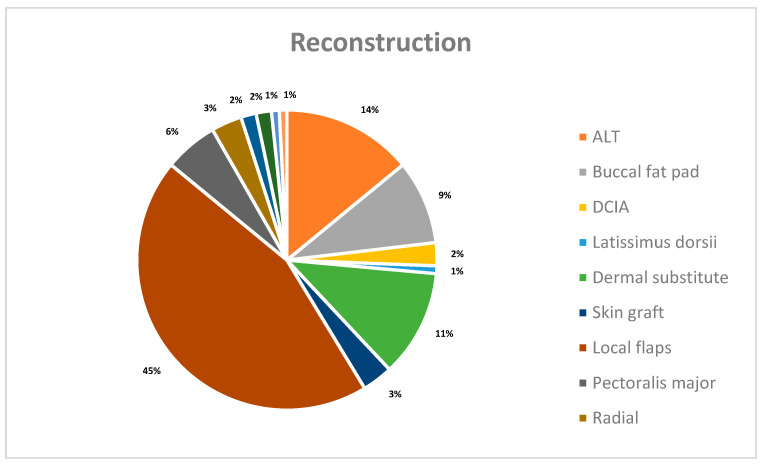
Free flap revascularisation procedures in 27 patients and their relative percentages.

From a reconstructive point of view, the majority of patients (54 (44.6%)) underwent reconstruction with local flaps; dermal substitutes were used in 14 (11.6%) cases; buccal fat pads in 11 (9.1%); skin grafts in 4 (3.3%); and in 10 patients, pedicled flaps were used, including 7 (5.8%) pectoralis major flaps, 2 (1.7%) supraclavicular flaps, and 1 (0.8%) temporalis muscle flap. Finally, free revascularised flaps were used in 27 patients: 17 (14%) anterolateral thigh (ALT) flaps, 4 (3.3%) forearm flaps, 2 (1.7%) chimeric scapula flaps, and in 3 cases (2.5%), a DCIA flap (Figure 1).

Histologically, 59 cases (48.8%) were staged as T1, and 62 (51.2%) as T2; 97 patients (80.2%) were confirmed as N0 neck, 10 patients as N1 (8.3%), 1 patient as N2a (0.8%), 8 patients as N2b (6.6%), 2 patients as N2c (1.7%), and 3 patients as N3b (2.5%). Regarding grading, 18 patients (14.9%) were G1, 85 patients (70.2%) were G2, and another 18 patients (14.9%) were G3. A total of 120 patients (99.2%) had R0 resection with healthy margins, while only 1 patient had R1 (0.8%). The mean DOI was 4.8 mm (range 1–9 mm), while the mean nodal yield for neck dissection was 38.56 lymph nodes per patient (Table 2).

**Figure 2:**
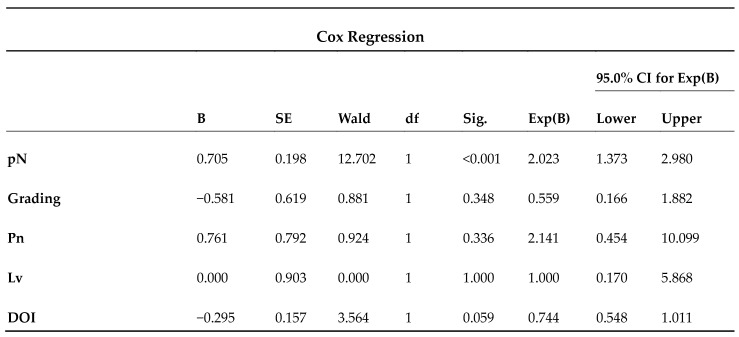
Cox proportional hazards regression. G (grading), Pn (perimeter invasion), LV (lymphovascular invasion), and DOI (depth of invasion) showed no statistically significant effect on survival (*p* > 0.05). pN (locoregional lymph node staging) showed a statistically significant effect on survival (*p* < 0.05). For this factor, Exp(B) > 1 and confidence interval (CI) entirely > 1 indicate an increased risk on the predictor and therefore a negative impact on survival.

Patients were followed up for an average of 45 months. At the end of data collection, 96 patients (79.3%) were alive, of whom 4 had disease (AWD). Further, 25 (20.6%) were dead; 20 (16.5%) died with disease (DWD), and 5 (4.1%) died without disease (DWoD). The results of the Cox hazard proportional regression showed a non-significant effect of Pn, LV, and DOI (*p* > 0.05) on survival, while pN staging was statistically significant (*p* < 0.05) with a strong negative impact on survival (Exp B 2.023; CI 95% 1.373–2.980) (Figure 2).

**Figure 3:**
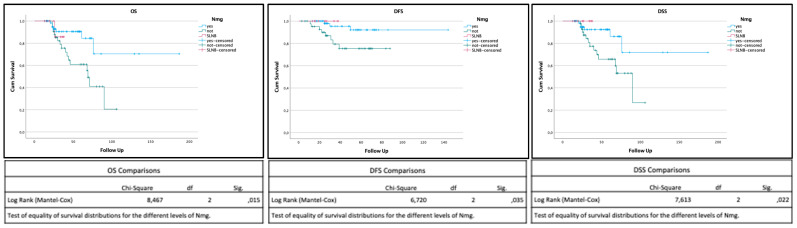
Kaplan–Meier curves for overall survival (OS), disease-specific survival (DSS), and disease-free survival (DFS). The OS of patients who received END (blue) and SLNB (purple) was significantly higher than that of patients who received FU (green) (*p* < 0.05). The same results were obtained for DSS and DFS (*p* < 0.05), leading to the rejection of the null hypothesis.

Survival analysis of Kaplan–Meier curves using the log-rank test showed a statistically significant difference in overall survival (OS), disease-specific survival (DSS), and disease-free survival (DFS) between patients who underwent END and SLNB and those who underwent FU (*p* < 0.05), leading to the rejection of the null hypothesis (Figure 3).

## 4. Discussion

Oral cancer is defined as a malignant neoplasm of the oral cavity and may involve several sites, such as the buccal mucosa, floor of the mouth, tongue, alveolar ridges, retromolar trigone, hard palate, and inner part of the lips. Although it has a significant incidence in Caucasian countries, its incidence is highest in Asian countries due to the widespread use of chewing tobacco. Other important risk factors include smoking, alcohol consumption, and socio-economic–cultural conditions, which often correlate with poor oral hygiene [10]. However, oral cancers currently rank 16th worldwide in terms of the number of cases, with an annual incidence of 377,000 new cases and approximately 178,000 deaths [11,12]. Of these, 90% are histologically related to squamous-cell carcinoma [13,14]. All oral squamous-cell carcinomas (OSCCs) are staged using the AJCC TNM (8th edition) system [3]. According to this staging system, early-stage OSCC (T1-2N0) accounts for 40–50% of new OSCC diagnoses. It is also important to distinguish between clinical and pathological staging. An early-stage OSCC newly diagnosed as cT1/T2N0 can easily progress to stage IV disease in the presence of a single lymph node metastasis on final histological report, with the consequent need for adjuvant therapies and a reduced prognostic outcome of up to 50% [15]. Furthermore, in this patient population, the percentage of occult metastases in a clinically N0 neck varies up to 30% of cases; therefore, the therapeutic management of the neck in early-stage T1/T2 OSCC is of paramount importance [16]. Occult metastases are defined as lymph node metastases that are not initially detected by neck palpation and imaging but are detected by histological examination, as micrometastases larger than 0.2 mm and smaller than 2.0 mm after neck dissection, or as metastases that appear as delayed regional recurrences during follow-up [17,18,19]. The management of the clinically N0 neck represents a long-standing topic of debate within the scientific literature. The principal therapeutic procedures described are END, FU with a policy of surveillance and eventual therapeutic neck dissection in the event of metastatic disease, and SLNB. The decision to perform FU in cN0 cases was based on the assumption that the neck can be treated therapeutically when and if patients develop locoregional metastases in the neck. Adherence to this principle ensures that patients who would not have developed metastases in the neck are spared the associated morbidity. It can be reasonably deduced that 80% of patients with N0 neck disease who undergo elective neck dissection will have received unnecessary neck treatment with its associated morbidity. Conversely, a strategy of observation carries the risk of occult metastases progressing to potentially incurable disease [17,20]. This principle has been the subject of considerable debate, with several studies in the literature indicating that, despite a higher incidence of adverse effects, END is associated with superior outcomes in terms of overall survival (OS), disease-free survival (DFS), and disease-specific survival (DSS) [16,21,22]. Over the past two decades, sentinel lymph node biopsy has emerged as a middle ground between these two opposing approaches [23,24]. Derived from breast surgery, SLNB is based on the analysis of the first lymphatic drainage station of the neoplasm. Neck dissection is only performed if the sentinel lymph node is positive; if the result is negative, no further treatment of the neck is necessary and neck dissection can be avoided. The aim of the procedure is therefore to reduce invasiveness, morbidity at the anatomical site, and healthcare costs [24,25,26,27]. One of the most important multicentre studies published to date is SENT [28], which demonstrates the oncological safety of the method in terms of morbidity, OS, and DSS. Although its conclusions are encouraging, the results need to be carefully analysed. Of the 415 patients enrolled in the study, 1342 sentinel lymph nodes (SLNs) were harvested, with an average of 3.2 lymph nodes per patient and a specified range of 1 to 10 lymph nodes. Considering that a neck dissection is considered functional for this purpose if at least 18 lymph nodes are removed [29,30], these data emphasise that SLNB is not yet standardised and the minimally invasive purpose varies from centre to centre and operator to operator. In addition, the false-negative rate (FNR) was 14% and the lymph node positive rate was 23%. This percentage of patients therefore underwent a second anaesthetic and a second surgical procedure. On the other hand, the study showed bilateral lymphatic drainage in 10% of cases and even contralateral to the lesion in 2.4% of cases. In these patients, scintigraphy was useful because in lateralised oral lesions, the patient would probably have undergone only ipsilateral neck dissection. Similarly, the work of Ionna et al. showed the results after 20 years of sentinel lymph node procedures performed at a single centre for T1/T2 squamous-cell carcinoma of the tongue. In their cohort of 122 patients, the average SN per patient was more homogeneous than in the multicentre study and was 1.4 lymph nodes per patient, with 24.6% positive sentinel nodes [31]. Although our patient cohort was derived from a single institution through a single-centre study, the sample is certainly representative. If we compare our study with the previously presented studies, it is easy to see how our 10-year cohort is equivalent to the 20-year cohort of the previously mentioned study and represents one-third of the cohort obtained from the international multicentre SENT trial over 5 years, with more than 15 centres recruited. Recently, Al-Moraissi et al. presented a meta-analysis on this topic. The authors looked at data from 1858 patients with a heterogeneous sample comprising 925 cases of END, 631 of FU, and 302 of SLNB. There was a statistically significant difference in OS and DFS in favour of END and SLNB compared to FU, while there was no statistically significant difference in OS and DFS between END and SLNB. On closer analysis of the surgical approach data, END outperformed SLNB. However, there is still a lack of data on SLNB in the literature, as well as data on morbidity, quality of life, and health care costs [32]. Similarly, the data from our study support those just mentioned. Cox regression highlighted the central importance of locoregional staging of N as a negative prognostic factor for survival (Figure 2). For the latter treatment, the null hypothesis of the oncological non-inferiority of FU was rejected, as a statistically significant difference was observed between END and SLNB for OS, DSS, and DFS (Figure 3). In any case, the EACMFS position paper by Vassiliou et al. is currently supported by the authors [16]. Although SLNB is an oncologically safe method and is included in the current and most recent treatment guidelines for oral squamous-cell carcinoma, it is important to exercise caution when selecting cases for SLNB, as approximately 25–30% of patients who undergo SLNB and are subsequently classified as SLNB+ experience a second surgical procedure and delayed adjuvant treatment. This is because they are definitively staged as N1. Given the correlation between DOI and a higher probability of metastasis, as well as the anatomical location of T, which also represents a risk factor for the success of SLNB, particularly the floor of the mouth (FOM), the authors currently utilise SLNB primarily in T1/T2 cases of the lateral tongue with a more exophytic growth pattern and no involvement of the posterior third. In cases of OSCC T1/T2 involving the posterior third of the tongue, FOM, alveolar ridge, and midline, the authors currently prefer END. However, other centres perform sentinel lymph node biopsies in all early-stage cases. From a reconstructive point of view, although the majority of early-stage carcinomas are reconstructed with local flaps, as was the case in our cohort of patients, revascularised free flaps represent an important therapeutic option in cases where functional reconstruction is desired, and especially in cases where the oro-cervical diaphragm is disrupted using the pull-through techniques for compartmental resections [33,34,35,36,37]. In summary, when analysing the data presented in our study and those reported in the literature, the clearest finding is the rejection of the null hypothesis that FU is oncologically non-inferior to other methods. Both END and SLNB are valid treatment options. Lai and colleagues are currently conducting a multi-centre randomised trial to try to answer the questions we are still asking and to define a gold standard for treatment. The main objectives of the study are to determine whether the DSS of patients treated with SLNB is inferior to that of patients treated with END and whether shoulder function is superior in patients treated with SLNB compared with END [9]. Despite the promising nature of SLNB, concerns remain regarding its efficacy. It is accurate to conclude that the technique has the potential to reduce healthcare costs, although this is contingent upon the duration of the patient’s hospitalisation. Furthermore, the financial implications of the lymphoscintigraphy procedure and the requisite equipment, including the use of a probe to locate the lymph node, must be taken into account. Furthermore, in comparison to END, it is a less reproducible technique, particularly in developing countries, where there may be greater challenges in establishing a nuclear medicine department capable of performing lymphoscintigraphy in collaboration with the surgeons responsible for the case. Conversely, the procedure has the potential to yield diagnostic insights, as lymphoscintigraphy can detect ‘unconventional lymphatic drainage patterns’ that may alter the course of treatment. However, as also indicated in Al-Moraissi’s systematic review [32], these data are still lacking from the existing literature. In our department, SLNB is a technique that is employed in specific cases. These cases include tumours of the lateral margin of the tongue with exophytic growth, where microsurgical reconstruction is not necessary. The floor of the mouth is considered the riskiest anatomical subsite in the context of SLNB due to its proximity to the neck, which can obscure the sentinel lymph node and make it difficult to locate through the probe, thereby reducing the invasiveness of the procedure. It should be emphasised that regardless of the location of the sentinel lymph node, an attempt is made to perform a mini-surgical approach along the lines of a possible END approach. It is important to remember that in the event of a positive sentinel lymph node or a false-negative result, the patient could undergo therapeutic neck dissection after the initial procedure for SLNB or during follow-up.

## 5. Conclusions

Early-stage T1-2N0 OSCC accounts for 50% of new OSCC diagnoses. Of these cases, 30% may be associated with occult metastases in the neck region. It is of utmost importance that accurate preoperative staging is carried out in order to ensure appropriate patient management. Among the challenging preoperative parameters to define, the depth of invasion is of particular importance, as it is significantly associated with an increased likelihood of occult metastases. From a therapeutic standpoint, this study yielded evidence that refuted the null hypothesis that FU is oncologically non-inferior to other surgical procedures. END and SLNB were found to be oncologically safe, more effective, and associated with better OS, DSS, and DFS. Sentinel lymph node biopsy was no less effective than elective neck dissection, although it is performed with limited indications in our centre. Further studies are needed to define the gold standard of therapy in the treatment of T1-2N0 early-stage OSCC.

## Figures and Tables

**Table 1:** Demographics, sites involved, and surgical management. END: elective neck dissection; FU: follow-up; SLNB: sentinel lymph node biopsy.

	Patients (%)
Men	65 (53.7)
Women	56 (46.3)
Mean Age	70.2
Site	
Tongue	53 (43)
Inferior alveolar ridge	17 (14)
Superior alveolar ridge	6 (5)
Lip	4 (3.3)
Floor of the mouth	14 (11.6)
Retromolar trigone	6 (5)
Cheek mucosa	17 (14)
Palate	4 (3.3)
T Management	
Resection	121 (100)
N Management	
END	68 (56.2)
FU	36 (29.8)
SLNB	11 (9.1)

**Table 2:** Main data from histology reports. T: pathological staging of primary tumour; N: pathological staging of locoregional lymph nodes; G: grading; R: resection with healthy margins; DOI: depth of invasion; N.Y. nodal yield.

	Patients (%)
T	
T1	59 (48.8)
T2	62 (51.2)
N	
N0	97 (80.2)
N1	10 (8.3)
N2a	1 (0.8)
N2b	8 (6.6)
N2c	2 (1.7)
N3a	0 (0)
N3b	3 (2.5)
G	
G1	18 (14.9)
G2	85 (70.2)
G3	18 (14.9)
R	
R0	120 (99.2)
R1	1 (0.8)
DOI	mean 4.8 (mm)
N.Y.	mean 38.5

## Data Availability

The data presented in this study are available on request from the corresponding author.

## Abbreviations

AWD: alive with disease; CT: computed tomography; DFS: disease-free survival; DOI: depth of invasion; DSS: disease-specific survival; DWD: dead with disease; DWoD: dead without disease; END: elective neck dissection; FU: follow-up for surveillance; LV: lymphovascular invasion; MRI: magnetic resonance imaging; OS: overall survival; OSCC: oral squamous-cell carcinoma; Pn: perineural invasion; SLNB: sentinel lymph node biopsy.
